# Teratoma: a set of teeth in the pelvis

**DOI:** 10.1590/0100-3984.2015.0034

**Published:** 2015

**Authors:** Thiago Krieger Bento da Silva, Guilherme Jaquet Ribeiro, Felipe Alba Scortegagna, Gláucia Zanetti, Edson Marchiori

**Affiliations:** 1 Department of Radiology, Hospital São Lucas – Pontifícia Universidade Católica do Rio Grande do Sul (PUCRS), Porto Alegre, RS, Brazil.; 2 Department of Radiology, Faculty of Medicine, Universidade Federal do Rio de Janeiro (UFRJ), Rio de Janeiro, RJ, Brazil.

**Figure 1:**
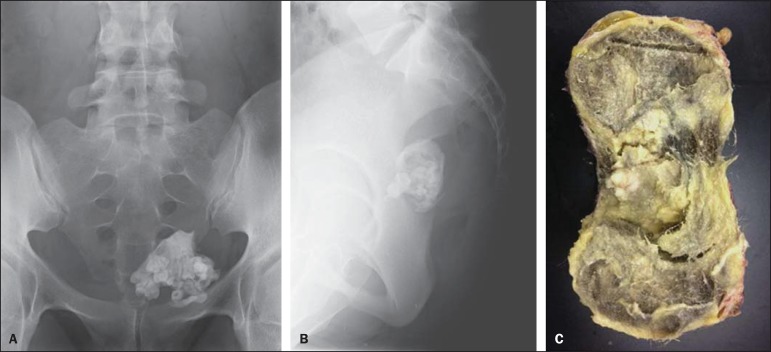
Frontal (**A**) and lateral (**B**) radiographic image of the pelvis showing a large calcified mass with multiple toothlike calcifications, indicative of a typical mature teratoma. Photo of the gross specimen (**C**) showing a well-circumscribed, encapsulated mass measuring 10.3 × 9.2 × 8.6 cm. The mass was filled with a yellowish viscous material, hair, and several tooth fragments.

*Dear Editor*,

A 25-year-old woman with neither history of trauma nor other previous medical history reported a 2-month history of low back pain. Physical examination revealed no significant abnormality. Conventional abdominal radiography showed the presence of a large, heterogeneous, calcified 10-cm mass in her left lower pelvis (Figures 1A and 1B). Pelvic ultrasonography (US) revealed a large heterogeneous mass containing internal hyperechoic areas with acoustic dirty shadowing in the left adnexal area, extending to the rectouterine pouch.

The patient underwent pelvic surgery with left adnexectomy. Macroscopically, the lesion measured 10.3 ×9.2 × 8.6 cm and was filled with a yellowish viscous material, hair, and several tooth fragments (Figure 1C). Analysis of histological specimens confirmed a mature teratoma containing mesodermal, endodermal, and ectodermal tissue.

A series of recent publications in the Brazilian radiological literature have evaluated the role of radiology in the study of abdominal tumors^(1-8)^.

The term “teratoma” comprises several histological types of tumor containing mature or immature tissue of the three germ cell layers: the ectoderm (skin, brain), mesoderm (muscle, fat), and endoderm (mucinous or ciliated epithelium)^(9-11)^. Mature teratoma is the most common benign ovarian tumor in women aged < 45 years. The clinical manifestations of ovarian teratoma range from an incidentally detected small mass to a malignantly transformed tumor associated with high mortality^(10)^. Most mature cystic teratomas are asymptomatic. Abdominal pain or other nonspecific symptoms occur in a minority of patients^(11)^.

At gross pathological examination, mature cystic teratomas are unilocular and frequently filled with sebaceous material and lined by squamous epithelium. Hair follicles, skin glands, muscle, and other tissues lie within the wall. A raised protuberance (Rokitansky nodule) usually projects into the cyst cavity. At any imaging modality, mature teratomas demonstrate a broad spectrum of findings ranging from purely cystic to mixed masses with components of all three germ cell layers, to noncystic masses composed predominantly of fat. Adipose tissue is present in 67– 75% of cases, and teeth are seen in 31%^(9-11)^.

Ovarian teratomas may cause various complications (e.g., torsion, rupture, malignant transformation, infection, autoimmune hemolytic anemia) with a wide spectrum of clinical and imaging features^(10)^. At conventional radiography, a typical mature teratoma appears as a large mass with fat opacity and/or multiple toothlike calcifications^(9)^. The most common US finding of an ovarian teratoma is a cystic mass with intratumoral fat and a densely echogenic tubercle (Rokitansky nodule) projecting into the cystic lumen^(10)^. Most mature cystic teratomas can be diagnosed by US, but such diagnosis is complicated by their diversity in appearance^(11)^. The diagnosis of mature cystic teratoma by computed tomography (CT) and magnetic resonance imaging (MRI) is fairly straightforward as such modalities are more fat sensitive. At CT, fat attenuation within a cyst, either with or without calcification in the wall, is diagnostic of mature cystic teratoma. Presence of fat is reported in 93% of cases and teeth or other calcifications in 56%. At MRI, the signal intensity of the sebaceous component of a teratoma is similar to that of retroperitoneal fat. Some hemorrhagic lesions may mimic this MRI appearance^(11)^.
